# Autofluorescence lifetime of gelatin-methacrylate hydrogels is sensitive to changes in crosslinking and post-gelation pH

**DOI:** 10.3389/fbioe.2026.1789773

**Published:** 2026-03-26

**Authors:** Marcos R. Rodriguez, Kyle P. Quinn

**Affiliations:** 1 Department of Biomedical Engineering, University of Arkansas, Fayetteville, AR, United States; 2 Department of Biomedical Engineering, Tufts University, Medford, MA, United States; 3 Arkansas Integrative Metabolic Research Center, University of Arkansas, Fayetteville, AR, United States

**Keywords:** autofluorescence, collagen, fluorescence lifetime, gelatin, hydrogel

## Abstract

**Introduction:**

There are limited tools to non-destructively evaluate the physicochemical properties of biomaterials in in vivo and in vitro settings. Here, we evaluate the autofluorescence of gelatin-methacrylate (GelMA) hydrogels through fluorescence lifetime imaging (FLIM) to assess its sensitivity to hydrogel photocrosslinking and post-gelation pH. GelMA is increasingly used as a biomaterial because it is highly tunable and has great biocompatibility.

**Methods:**

Using two-photon excitation, we identified optimal acquisition and analysis approaches to detect changes in the autofluorescence lifetime of gelatin. Through a combination of phasor analysis and multi-exponential modeling, we determined that a tri-exponential decay fit accurately captured multiple intrinsic fluorescent species in gelatin.

**Results:**

The mean lifetime ( and the relative contribution ratios of the lifetime species (A_1_/A_2_, A_1_/A_3_, and A_2_/A_3_) were sensitive to both crosslinking and microenvironmental pH. As hydrogel crosslinking increased following an increase in photoinitiator concentration from 0% to 0.50%, lifetime parameters, A_1_/A_3_ and A_2_/A_3_ were altered. Moreover, as hydrogels were submerged in buffers with pH values increasing from 5.8 to 9.0, lifetime parameters , A_1_/A_2_ and A_1_/A_3_ changed.

**Discussion:**

These results demonstrate that FLIM has potential in distinguishing between distinct alterations to the molecular microenvironment of biomaterials without the need for exogenous fluorophores. In addition, we demonstrate that lifetime changes associated with crosslinking are related to gelatin autofluorescence rather than any crosslink autofluorescence.

## 1 Introduction

Biomaterials have become ubiquitous in biomedical research and have aided in the translation of therapeutics to the clinic (Gammon et al., 2016; Tang et al., 2019). Hydrogels in particular have helped to advance drug delivery, tissue engineering, and biosensor research due to their hydrophilicity, biocompatibility, mechanical behavior and three-dimensional structure (Chai et al., 2017). These characteristics also allow hydrogels to be used to study cell-material and tissue-material interactions through cell encapsulation and *in vivo* implantation (Celikkin et al., 2022; Dang et al., 2022; Griveau et al., 2022). The development of synthetic and semi-synthetic hydrogels has also provided the means to increase tunability and exploit molecular binding, with semi-synthetic hydrogels still possessing innate biocompatibility (Panahi and Baghban-Salehi, 2019). As of late, semi-synthetic, photopolymerizable gelatin-methacrylate (GelMA) hydrogels have gained much interest (Cruz-Acuña et al., 2019; Xiao et al., 2019; Elkhoury et al., 2020; Dang et al., 2022; Kong et al., 2022). GelMA is made from the functionalization of collagen-derived gelatin with methacrylate groups, which allows it to exhibit the biodegradability, extracellular matrix mimicry and cellular binding motifs of native collagen while also being thermo- and pH-responsive (Anirudhan and Mohan, 2018; Osi et al., 2021). Since its inception, GelMA was developed as a highly biocompatible hydrogel that is chemically stable at physiological temperature following light polymerization for wound dressing applications (Van Den Bulcke et al., 2000). It has been used in studies involving wound healing, skin allografts, bioprinting, and drug delivery (Yin et al., 2018; Uehara et al., 2019; Yi et al., 2020; Yan et al., 2021). However, when evaluating complex cell-hydrogel interactions such as cellular infiltration, proliferation, hydrogel degradation, or matrix remodeling, destructive measurement techniques are typically used to monitor changes in hydrogel physicochemical properties.

Over the last few decades, various techniques have been explored and expanded upon for characterizing morphology, mechanical performance, molecular composition, and biocompatibility of hydrogels (Musilová et al., 2018; Onaciu et al., 2019; Raghuwanshi and Garnier, 2019; Abrego et al., 2022). Rheology is typically employed to evaluate hydrogel mechanical properties, such as the storage and loss modulus, while sweeping hydrogel temperature and deformation frequency (Diamantides et al., 2017; McNamara et al., 2021). Histology provides valuable microstructural information before or after *in vivo* implantation, but it requires the mechanical sectioning and staining of tissue and, like rheology, cannot be used to longitudinally monitor changes in hydrogel properties *in vivo* (Uehara et al., 2019). Nuclear magnetic resonance spectroscopy is routinely used to determine the degree of methacrylation of GelMA and can measure changes in cellular protein expression, but suffers from low spatial resolution (Nichol et al., 2010; Lee et al., 2017; Luchinat and Banci, 2018; Heckova et al., 2019; Chaudhary et al., 2022). Fluorescent proteins have been incorporated into hydrogels to monitor dynamics, but have limited photostability and can undergo proteolysis (Day and Davidson, 2009; O’Donnell et al., 2018). Incorporating fluorescent labels into a biomaterial can also complicate FDA approval and translation to the clinic. Therefore, there is a need for label-free, non-destructive characterization methods to evaluate dynamic changes in hydrogel properties both *in vivo* and *in vitro*. Gelatin and collagen can exhibit autofluorescence from amino acid residues such as tyrosine and phenylalanine that emit in the UV range, crosslinks such as pyridinoline and pentosidine, and complex advanced glycation end products (AGEs) that emit in the visible range (Hassan et al., 2025; Duconseille et al., 2016; Saletnik et al., 2023; Atzeni et al., 2022; Quinn et al., 2016; Kchaou et al., 2018; Risum et al., 2024). Thus, gelatin autofluorescence may be leveraged as a source of contrast for characterizing GelMA hydrogels.

Fluorescence lifetime imaging (FLIM) offers promise in characterizing dynamic changes in the properties of naturally fluorescent biomaterials as it can non-destructively measure changes in the microenvironment of a fluorophore. Fluorescence lifetime is the time between excitation of a sample and emission of an individual photon, and it is a natural property of any fluorophore. Through time-correlated single-photon counting (TCSPC), photon decay curves can be measured and fluorescence lifetimes can be calculated on a pixel-by-pixel basis (Kolenc and Quinn, 2019). If multiple lifetime species are present at a given pixel, a decay curve can be fit through a multi-exponential model to determine the relative amounts of each species. Because it is sensitive to the proportion of radiative and non-radiative transitions, FLIM is sensitive to protein binding, pH, viscosity, and temperature (Kuimova et al., 2008; Chen et al., 2014).

FLIM has utility in distinguishing between multiple lifetime species within a pixel based on their unique decay rates, which has extensive applications in diagnosing diseased cells and tissues by assessing metabolism through measurement of free- and protein-bound NADH and FAD lifetime (Skala et al., 2007; Meier et al., 2010; Stringari et al., 2011; Mazumder et al., 2013; Sameni et al., 2016; Stuntz et al., 2017; Jones et al., 2018; 2020; Kolenc and Quinn, 2019). Although cellular autofluorescence has been the focus of most label-free FLIM studies, the technique has also been used to characterize biomaterials. Studies have demonstrated that fluorescence lifetime correlates with hydrogel storage modulus and Young’s modulus, and is sensitive to biomaterial pH and drug release from polymeric nanoparticles (O’Donnell et al., 2018; Sherlock et al., 2018; Zhou et al., 2018). While gelatin autofluorescence has been exploited to evaluate surface binding, there is minimal reporting of the utility of using its autofluorescence to characterize hydrogel properties (Farbod et al., 2016).

The goal of this study was to explore the use of FLIM to observe changes in GelMA hydrogel properties. We hypothesized that FLIM may be capable of delineating changes in GelMA crosslinking and pH based on the natural fluorescence of gelatin. To this end, we varied the crosslinks present in GelMA through photoinitiator concentration and adjusted pH by submerging hydrogels in buffers at different pHs. The hydrogels then underwent two-photon excited fluorescence (TPEF) imaging to identify excitation and emission wavelengths that produce optimal GelMA autofluorescence signal. TPEF allows for intrinsic depth sectioning, more efficient light collection, deeper tissue penetration, and minimal photodamage compared to confocal microscopy, which makes it advantageous for future 3D *in vivo* and *in vitro* imaging studies involving GelMA. After identifying the wavelengths that produced peak GelMA autofluorescence, we used TPEF lifetime imaging to monitor hydrogel properties. Through phasor analysis of fluorescence lifetime, we identified three lifetime species and used a tri-exponential decay model to detect changes associated with crosslinking and pH. This method of measuring time-resolved changes in gelatin autofluorescence may have broad applicability in non-destructively evaluating dynamic physicochemical changes of gelatin-based biomaterials.

## 2 Materials and methods

### 2.1 Hydrogel preparation with varying crosslinking degrees

Methacrylated gelatin (Advanced Biomatrix, Carlsbad, CA, United States) was dissolved in 1X PBS at 37 °C while the photoinitiator, Irgacure 2,959 (Advanced Biomatrix, Carlsbad, CA, United States) was dissolved in 1X PBS at 70 °C (Loessner et al., 2016). The photoinitiator solution was then added to 5.0% (w/v) GelMA solutions to yield four different photoinitiator concentrations of 0%, 0.05%, 0.25% and 0.50% (w/v). Irgacure 2,959 undergoes Norrish type I cleave and dissociates in benzoyl and ketyl radicals upon UV exposure (Yue et al., 2015; Atef et al., 2025; Dogan et al., 2026). These radicals react with the carbon-carbon double bonds of the methacryloyl groups and initiate chain polymerization through propagating radicals that result in the covalently crosslinked polymer network of the hydrogels (Yue et al., 2015). To photocrosslink GelMA with the photoinitiator, 200 µL of the different GelMA-photoinitiator solutions were warmed to 37 °C, transferred to glass-bottom 35 mm dishes and exposed to 306 nm UV light at an intensity of 4.4 mW/cm^2^ for 45 s to achieve a total UV dose of 198 mJ/cm^2^ (Nichol et al., 2010; Loessner et al., 2016).

### 2.2 Hydrogel preparation with varying pH

Following the steps from the previous section, a solution was prepared containing 5.0% (w/v) GelMA and 0.50% (w/v) photoinitiator in 1X PBS. Considering that buffers only stabilize pH over specific pH ranges, we selected PBS as the buffer from acidic to neutral pH and tris-EDTA (TE) from neutral to basic pH. Thus, 1X PBS buffers were prepared at pH values of 5.8 and 7.4, while tris-EDTA (TE) buffers were prepared at pH values of 7.4 and 9.0 (Yang et al., 2008; Stoll and Blanchard, 2009; Suman et al., 2012). This allowed us to evaluate the change in autofluorescence lifetime when GelMA is treated with different buffers at the same pH, as well as the effect that modifying the pH of the buffers would induce. The buffers were pH-adjusted with hydrochloric acid (HCl) and sodium hydroxide (NaOH). After the hydrogels were developed by photocrosslinking GelMA with UV light for 45 s, 1X PBS and TE buffers were transferred to the cell culture plates ensuring that the hydrogels were fully submerged and treated for 24 h. Once the 24-h treatment cycle finished, the excess buffer was decanted and removed with deionized water.

### 2.3 Two-photon excited fluorescence imaging and analysis of GelMA hydrogels

Signal from the GelMA hydrogels was obtained using a two-photon excited fluorescence (TPEF) microscope (Bruker Investigator, Billerica, MA, United States) outfitted with a 20x, 1.0 NA water-immersion objective (Olympus, Tokyo, Japan), 3 GaAsP photomultiplier tubes (PMTs) (H10770PA-40; Hamamatsu, Shizuoka, Japan) spanning 380–550 nm (FFO1-440/SP-25; IDEX Health and Science, West Henrietta, New York; ET460/40m-2p, ET525/50m-20; Chroma, Bellows Falls, Vermont), and a Ti:Sapphire laser (MaiTai or Insight X3; Spectra Physics, Mountain View, CA, United States). Excitation spectra of the hydrogels were collected at 720, 740, 755, 780, 800, 820, 840, 855, 880, and 900 nm at three distinct locations. At each excitation wavelength, hydrogel fluorescence emission intensity was acquired in green (525 ± 25 nm), blue (460 ± 20 nm), and UV (≤430 nm) range. Fluorescence intensity was normalized by PMT gain and laser power as described in previous studies (Jones et al., 2018; 2020). After normalization, the pixels across the intensity images were averaged to calculate a mean intensity value for each image location.

### 2.4 Autofluorescence lifetime imaging of GelMA hydrogels

Autofluorescence life time images were obtained using time-correlated single-photon counting (TCSPC) hardware (SPC-150; Becker-Hickl GmbH, Berlin, Germany) at three to five distinct locations from each hydrogel. Lifetime images were acquired at 840 nm excitation and 525 ± 25 nm emission over an integration time of 120 s. Lifetime images were processed using SPCImage v8.0 (Becker and Hickl GmbH, Berlin, Germany), and an instrument response function (IRF) for the system was obtained using the second harmonic signal of urea crystals and used to deconvolve the measured FLIM decay curves.

### 2.5 Phasor and multi-exponential analysis of lifetime images

Fit-free phasor analysis was performed on the lifetime images, where the equations below were used to transform the photon decay at every pixel to points in a polar plot (Digman et al., 2008). Representing the photon decay in phasor space allows for the number of different lifetime species present in images to be identified based on the shape of the phasor coordinate distribution (Digman et al., 2008; Stringari et al., 2011).

$$g_{i,j}\left(\omega \right)=\frac{\int_{0}^{\infty }I_{i,j}\left(t\right)\cos\left(\omega t\right)dt}{\int_{0}^{\infty }I_{i,j}\left(t\right)\text{dt}}$$

$$s_{i,j}\left(\omega \right)=\frac{\int_{0}^{\infty }I_{i,j}\left(t\right)\sin\left(\omega t\right)dt}{\int_{0}^{\infty }I_{i,j}\left(t\right)\text{dt}}$$

Although phasor analysis offers the ability to determine the number of lifetime species present in an image, multi-exponential fitting of photon decay is commonly employed to determine the relative contributions of the lifetime species present at a given pixel. Using the phasor analysis as a starting point, we evaluated different multi-exponential models using the equation below to best discriminate the lifetime species observed through FLIM. The model fits that exhibited an average 0.5 < X^2^ < 1.5 were considered appropriately fit. Additionally, the images were spatially binned ten times to maximize fluorophore signal while maintaining an appropriate X^2^ value.

$$\frac{I\left(t\right)}{I_{0}}=\sum A_{i}\exp\left(-t/\tau_{i}\right)$$

Lifetime parameters were averaged across all pixels within each FLIM image, and ratios of each lifetime species relative to each other were calculated. Using all lifetime parameters, the weighted average of the lifetimes (i.e., the mean lifetime, *τ*_m_) was calculated using the equation below and averaged for each FLIM image as well.

$$\tau_{m}=\sum A_{i}\;*\;\tau_{i}$$

### 2.6 Statistical analysis

Mixed-effects ANOVAs fitted *via* restricted maximum likelihood (REML) were used to compare FLIM metrics. Hydrogel crosslinking levels and buffer pH were considered a fixed effect, while image locations were nested within individual hydrogels as random effects. Statistical significance was defined when *p* < 0.05 which is denoted by asterisks in the figures. Statistical tests were performed using JMP 18.2.2 (Cary, NC, United States). Bar graphs in figures represent group means and error bars represent standard deviations.

## 3 Results

### 3.1 Autofluorescence signal of GelMA hydrogels

**FIGURE 1:**
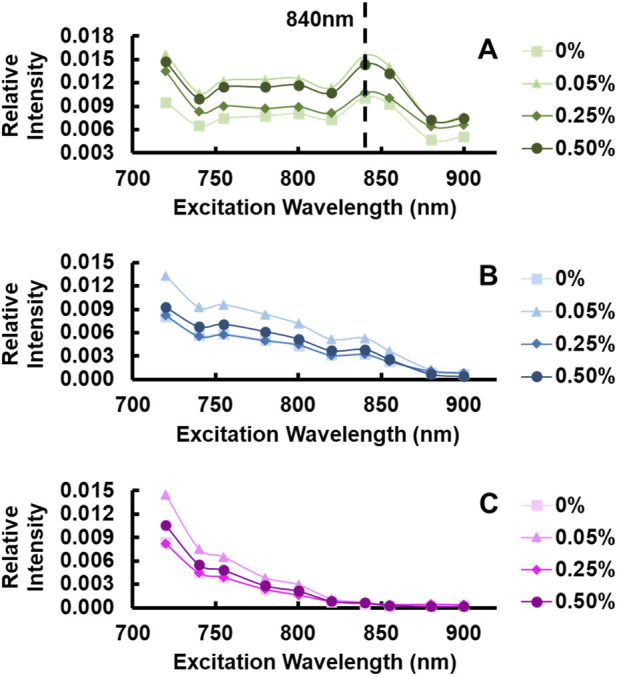
GelMA hydrogel fluorescence spectra at distinct photoinitiator concentrations (n = 4–6 per photoinitiator concentration) collected between **(A)** 500–550 nm (Green), **(B)** 440–480 nm (Blue), and **(C)** ≤430 nm emission wavelengths.

The GelMA hydrogels exhibited a weak but detectable autofluorescence signal that was strongest at 525 ± 25 nm emission. The autofluorescence intensity appeared to be independent of the degree of crosslinking (Figure 1). Within this green emission range, a clear autofluorescence intensity peak was observed between 800–855 nm excitation for all hydrogels (Figure 1A). This intensity peak was not clearly observed in the 460 ± 20 nm (Figure 1B) and ≤430 nm (Figure 1C) spectral ranges. While the detected signal increased in all emission channels at lower excitation wavelengths approaching 720 nm, it was determined that this signal was not autofluorescence, but rather a very small fraction of reflected excitation light reaching the detectors (Figure 1). Based on these observations, all FLIM acquisition occurred at 840 nm excitation and 525 nm emission.

Although autofluorescence often occurs due to crosslinking, the GelMA hydrogels exhibited an identical spectral shape on all channels independent of crosslinking degree (Figure 1) (Sherlock et al., 2018). Additionally, the photoinitiator was tested for autofluorescence after being dissolved in 1X PBS and did not exhibit any detectable signal. Moreover, the solution containing GelMA without the photoinitiator had an identical spectral shape and peak excitation-emission configuration as hydrogels with increasing levels of photoinitiated crosslinks (Figure 1A).

### 3.2 Phasor coordinate spread of GelMA hydrogel photon decay

**FIGURE 2:**
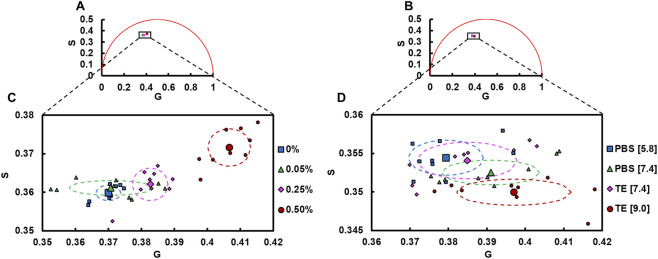
Phasor analysis of GelMA hydrogel lifetime at different **(A)** crosslinking degrees and using different **(B)** buffer treatments (n = 9 per photoinitiator concentration and buffer treatment). Focusing on the area occupied by the hydrogels’ phasor coordinates demonstrates different coordinate shifting due to **(C)** crosslinking when compared to **(D)** pH. Group averages are represented as large markers and individual image averages as small markers. Ellipses represent standard deviations for each group.

Phasor plots were developed of GelMA lifetimes acquired at different crosslinking degrees (Figure 2A) and pHs (Figure 2B). Focusing on the area occupied by the hydrogels’ phasor coordinates indicated that the coordinates shifted positively in the g- and s-axes as the crosslinking degree increased (Figure 2C). The average g and s coordinates of hydrogels with the highest (0.50%) photoinitiator concentration (g = 0.407 ± 0.006; s = 0.372 ± 0.005) were significantly greater than lower concentrations (p < 0.0001). Furthermore, hydrogels prepared with 0.25% photoinitiator concentration had an average g coordinate (0.383 ± 0.005) that was greater than that of the 0.05% (0.371 ± 0.013) and 0% (0.370 ± 0.004) samples (p ≤ 0.0135). The phasor plot among varying pH levels was distributed differently, with a positive shift in the g-axis and a negative shift in the s-axis as the pH of the buffers increased (Figure 2D). Hydrogels treated with TE at a pH of 9.0 had an average g coordinate (0.397 ± 0.015) greater than those treated with PBS at a pH of 5.8 (0.379 ± 0.010) and had an average s coordinate (0.350 ± 0.004) less than those treated with PBS at a pH of 5.8 (0.354 ± 0.002) and TE at a pH of 7.4 (0.354 ± 0.002) (p ≤ 0.0278). These shifts in the phasor plot suggest that there are multiple lifetime species present in GelMA.

### 3.3 Fitting of photon decay with exponential models

**FIGURE 3:**
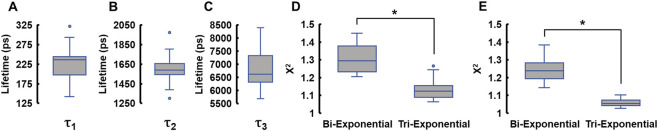
**(A-C)** Lifetime values obtained through tri-exponential fitting of the subset (n = 24 images; data from all groups was used). After fixing median lifetime values obtained from subset analysis, the X^2^ of the total dataset was closer to one when using a tri-exponential model regardless of **(D)** crosslinking degree and **(E)** buffer pH (n = 36 hydrogels per exponential model). Boxes correspond to the first and third quartiles, and the horizontal lines across the boxes correspond to the medians. Whiskers extend to the full range of the data excluding outliers, and circular markers represent outliers. * represents p < 0.05.

The distribution of lifetime decays within the phasor plots suggested that three lifetime species may be contributing collectively to a sensitivity to crosslinking and pH. Bi-exponential fitting of lifetime decay is far more common than tri-exponential fitting in autofluorescence lifetime analysis of biological tissues due to concerns of overfitting data with limited signal-to-noise ratios (Köllner and Wolfrum, 1992; Esposito and Wouters, 2004). To improve fitting with a tri-exponential model, we sought to reduce the degrees of freedom of the model to below that of a bi-exponential model, which involves three independent fit parameters. Using a subset of the data (24 lifetime images) that spanned all groups, an initial unconstrained tri-exponential fit was performed. Relatively consistent time constants were collected from the fits of these data, and median values (*τ*_1_ = 236 ps; *τ*_2_ = 1,588 ps; *τ*_3_ = 6,613 ps) were obtained and fixed for subsequent fitting with the entire dataset (Figures 3A–C). This approach allows for only two independent unfixed parameters, and despite fewer degrees of freedom when compared to a bi-exponential model, this modified tri-exponential fitting (X^2^ = 1.13 ± 0.08 for crosslinking dataset; X^2^ = 1.06 ± 0.02 for pH dataset) resulted in improved X^2^ values compared to the unconstrained bi-exponential fit (X^2^ = 1.30 ± 0.08 for crosslinking dataset; X^2^ = 1.24 ± 0.07 for pH dataset) (Figures 3D,E).

### 3.4 Effect of varying crosslinking degree on autofluorescence lifetime

**FIGURE 4:**
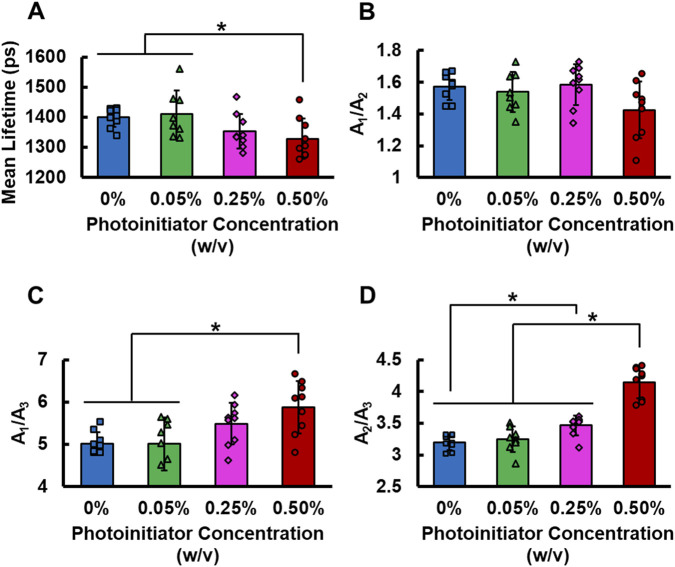
**(A)** Mean fluorescence lifetime **(B)** ratio of contribution of *τ*_1_ species to *τ*_2_ species **(C)** ratio of *τ*_1_ species to *τ*_3_ species, and **(D)** ratio of *τ*_2_ species to *τ*_3_ species of GelMA hydrogels at varied degrees of crosslinking (n = 9 per photoinitiator concentration). Error bars represent standard deviations for each group. * represents p < 0.05.

Hydrogels produced a shorter mean fluorescence lifetime when photoinitiator concentration was increased (Figure 4A), suggesting an inverse relationship between crosslinking and lifetime. Specifically, GelMA hydrogels imaged with 0.50% photoinitiator displayed a shorter mean fluorescence lifetime (1,328.78 ± 66.55 ps) than that of hydrogels crosslinked with 0.05% (1,409.90 ± 58.02 ps) and of hydrogels without the presence of the photoinitiator (1,399.06 ± 31.70 ps) (p ≤ 0.0373; Figure 4A). Although the presence of the shortest lifetime component (A_1_) relative to A_2_ was not significantly affected (p = 0.0569) by variations in crosslinking degree (Figure 4B), both A_1_/A_3_ and A_2_/A_3_ were significantly increased as the crosslinking in the GelMA hydrogels increased (p ≤ 0.0008) (Figures 4C,D). Hydrogels crosslinked with 0.50% photoinitiator exhibited a significantly higher A_1_/A_3_ ratio (5.88 ± 0.62) compared to those crosslinked with 0.05% photoinitiator (5.01 ± 0.62) or no photoinitiator (5.02 ± 0.26) (p ≤ 0.0060; Figure 4C). Differences were also observed in the A_2_/A_3_ ratio among crosslinking levels as hydrogels crosslinked with 0.50% photoinitiator had a significantly larger A_2_/A_3_ (4.15 ± 0.25) than the rest of the groups (p < 0.0001; Figure 4D). In addition, hydrogels crosslinked with 0.25% photoinitiator exhibited a significantly larger A_2_/A_3_ (3.46 ± 0.15) than those formulated with 0% photoinitiator (3.20 ± 0.12) (p = 0.0242; Figure 4D). Together, these results indicate that changes in GelMA crosslinking levels cause a relative reduction in the contribution of the longest lifetime component of gelatin.

### 3.5 Effect of altering pH on autofluorescence lifetime

**FIGURE 5:**
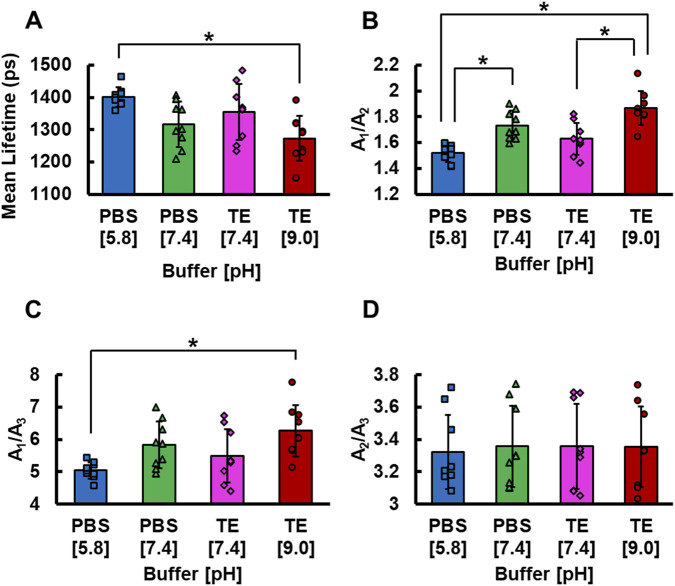
**(A)** Mean fluorescence lifetime **(B)** ratio of contribution of *τ*_1_ species to *τ*_2_ species **(C)** ratio of *τ*_1_ species to *τ*_3_ species, and **(D)** ratio of *τ*_2_ species to *τ*_3_ species of GelMA hydrogels after treatment with buffers at distinct pHs (n = 9 per photoinitiator concentration). Error bars represent standard deviations for each group. * represents p < 0.05.

GelMA hydrogels mean lifetime decreases as the pH of the buffers increase (p = 0.0025; Figure 5A). Hydrogels submerged in PBS at a pH of 5.8 (1,401.27 ± 29.65 ps) exhibited a significantly longer mean fluorescence lifetime (p ≤ 0.0017) than those submerged in TE at a pH of 9.0 (1,273.04 ± 70.17 ps) (Figure 5A). Differences in fluorescence lifetime associated with pH were even more apparent through the A_1_/A_2_ ratio (p < 0.0001; Figure 5B). Specifically, hydrogels treated with TE at a pH of 9.0 (1.87 ± 0.13) exhibited a higher A_1_/A_2_ (p ≤ 0.0005) than those treated with PBS at a pH of 5.8 (1.52 ± 0.07) and TE at a pH of 7.4 (1.63 ± 0.12). In addition, hydrogels treated with PBS at a pH of 7.4 also displayed a higher A_1_/A_2_ (1.73 ± 0.11) when compared to those treated with PBS at a pH of 5.8 (p = 0.0017). The A_1_/A_3_ ratio was also influenced by microenvironmental pH (p = 0.0052; Figure 5C). This, however, was only observed between hydrogels treated with PBS at a pH of 5.8 (5.04 ± 0.25) and those treated with TE at a pH of 9.0 (6.27 ± 0.79) (p = 0.0034; Figure 5C). Unlike crosslinking levels, which significantly altered the A_2_/A_3_ ratio, differences in microenvironmental pH were not detectable with this ratio of lifetime species (Figure 5D). Of note, there were no observable differences in any lifetime parameter between hydrogels treated with PBS at a pH of 7.4 and hydrogels treated with TE at a pH of 7.4, suggesting that changes in lifetime were not influenced by the buffer type but rather by the pH of the buffers.

## 4 Discussion

Gelatin-methacrylate has increasingly been utilized for bioengineering applications, and nondestructive methods to test, validate, and monitor hydrogel properties will be of great importance for translating GelMA hydrogels to various clinical applications (Kurian et al., 2022). Although studies have reported on the autofluorescence of collagen and gelatin, studies reporting the autofluorescence of GelMA and its sensitivity to relevant physiochemical changes are lacking (Brigo et al., 2017; Elkhoury et al., 2020; Poologasundarampillai et al., 2021). In this study, we demonstrate that GelMA exhibits detectable autofluorescence primarily in the green emission range following two-photon excitation at 820–855 nm and that the lifetime of this autofluorescence is altered in different ways when its crosslinking levels and pHs are modified. Considering the relatively long wavelengths that lead to peak gelatin autofluorescence (840 nm excitation/525 nm emission; Figure 1), the likely source of the autofluorescence is AGEs that naturally accumulated in collagen and may also be produced in during gelatin processing (Zoumi et al., 2002; Quinn et al., 2016). Studies have also demonstrated sensitivity to crosslinks due to exposure to glutaraldehyde and formation of glycation products, which are known to produce fluorescent crosslinks (Fujimori, 1989; Zhu et al., 2017; Sherlock et al., 2018). However, an increase in photoinitiated crosslinks in GelMA does not produce an increase in TPEF intensity nor does it alter excitation spectra like other types of crosslinks. Moreover, the photoinitiator, Irgacure 2,959, did not exhibit detectable signal, and the same autofluorescence signatures were present in the absence of photo-crosslinking (Figure 1). Taken together, our results suggest that changes in the autofluorescence lifetime of GelMA associated with photocrosslinking are due to changes in the local microenvironment of fluorophores naturally present in gelatin. In addition, we should note that the average fluorescence intensity of the hydrogels is comparable to that observed from dermal collagen imaging at similar spectral bands (Rodriguez et al., 2026), indicating that this technique could be used to map out changes in GelMA properties with high resolution.

Fluorescence lifetime decays of collagen are typically fit to multi-exponential models due to the broad autofluorescence that is produced by collagen and other sources of fluorescence present, particularly as a result of crosslinking (Blackwell et al., 2008; Lutz et al., 2012; Manickavasagam et al., 2014; Fukushima et al., 2015). The broad autofluorescence has also led to the use of multi-spectral FLIM acquisition of collagen where the average lifetime is reported, and bi-exponential or tri-exponential modeling of the decay is routinely employed (Lutz et al., 2012; Fukushima et al., 2015; Ranjit et al., 2015; Sherlock et al., 2018; Vazquez-Portalatin et al., 2020). Phasor analysis, which is a fit-free approach, has utility in helping determine the number of fluorescent lifetime species present. Through our phasor analysis of GelMA lifetime decay (Figure 2), we determined that a two-component model of GelMA lifetime was inadequate and that, despite a reduction in the degrees of freedom, a tri-exponential model with fixed time constants provided a superior fit (Figures 3D,E). Nevertheless, we should note that the average X^2^ value of the bi-exponential fits were within ranges considered acceptable and that prior studies have used lifetime parameters to represent weighted averages of multiple species (Blacker et al., 2014; Fukushima et al., 2015). It should further be noted that biomaterials such as gelatin, GelMA or collagen display broad autofluorescence and may even have additional lifetime species that cannot be resolved with current acquisition and analysis methods.

There are conflicting results found in the literature when evaluating the relationship between collagen crosslinking and autofluorescence lifetime. Some studies have found that increases in crosslinking will increase collagen lifetime while others have found that the lifetime will decrease as more crosslinks are present (Lutz et al., 2012; Manickavasagam et al., 2014; Fukushima et al., 2015; Sherlock et al., 2018; Vazquez-Portalatin et al., 2020). This is likely because the relationship between crosslinking degree and fluorescence lifetime depends on the type of crosslinking and whether the crosslinks formed are naturally fluorescent (Sherlock et al., 2018). Our results highlight that, even if the crosslinks themselves do not fluoresce, the microenvironment of fluorophores naturally present in collagen and gelatin can be altered. It is possible that viscoelastic changes produced by crosslinking could cause a change in lifetime (Kuimova et al., 2008). Our data indicates that a decrease in mean lifetime of GelMA is associated with increasing the levels of crosslinks. Although the relative contribution of short and long lifetime components may potentially be used as a marker of crosslinks present in GelMA (Figure 4C), A_2_/A_3_ is potentially a more sensitive parameter for this purpose (Figure 4D). Given that increased crosslinking is associated with greater hydrogel stiffness (Manickavasagam et al., 2014; Fukushima et al., 2015; Pepelanova et al., 2018; Sherlock et al., 2018; Vazquez-Portalatin et al., 2020), our FLIM outcomes may be indirectly sensitive to stiffness. This holds biological relevance when conducting *in vivo* and *in vitro* studies with GelMA as the stiffness of GelMA will determine the behavior of cells and tissues, and may change itself as the cells remodel the hydrogel (Wu et al., 2019; Martinez-Garcia et al., 2021; Ibañez et al., 2021). However, additional studies are required to definitively elucidate the relationships among autofluorescence lifetime, crosslinking levels, and hydrogel stiffness.

Variations in pH occur in native and engineered tissues as a result of cell metabolism and a variety of biological processes (Oh et al., 2009; Buckley et al., 2018). Furthermore, altering the pH of a hydrogel can affect surface tension, drug delivery kinetics, printability, and cellular behavior (Paradise et al., 2011; Diamantides et al., 2017; Zhou et al., 2018; Vigata et al., 2021). Our results suggest that FLIM-based measurements of GelMA may be sensitive to these pH-related changes. For example, gelatin solutions have been shown to experience an increase in surface tension when their pH is increased from 5 to 10 (Johlin, 1930). Fluorescence lifetime also has potential in monitoring the extracellular pH of gelatin-encapsulated cells when they release molecules such as lactate, which causes extracellular acidification (De La Cruz-López et al., 2019). Skin wound pH is also known to fluctuate from four to 6.5, which may be reflected in the autofluorescence lifetime of a hydrogel (Tang et al., 2021). In our study, increasing the pH ultimately decreased the mean fluorescence lifetime of GelMA hydrogels (Figure 5A). This was predominantly the result of a increase in the relative contribution of the short 236 ps component (Figures 5B,C). Previous studies have had conflicting results regarding the relationship between pH and fluorescence lifetime as it seems to depend on the specific fluorescent molecule that is being evaluated (Lin et al., 2001; Ryder et al., 2001; Hanson et al., 2002; Nakabayashi et al., 2008; Ogikubo et al., 2011; Islam et al., 2013; Orte et al., 2013; Chen et al., 2014; O’Donnell et al., 2018; Zhou et al., 2018; Chacko and Eliceiri, 2019; Schmitz et al., 2021). Moreover, studies typically incorporate exogenous sources of fluorescence to monitor changes in pH rather than relying on endogenous fluorophores like gelatin (Lin et al., 2001; Ryder et al., 2001; Hanson et al., 2002; Nakabayashi et al., 2008; Orte et al., 2013; O’Donnell et al., 2018). Given that new exogenous dyes face various regulatory hurdles, our approach that relies on the natural fluorescence of gelatin offers an easier path to immediate clinical translation. In addition, since variations in crosslinking and pH were reflected in two distinct lifetime parameters (A_2_/A_3_ and A_1_/A_2_, respectively), our results suggest an ability to differentiate between distinct changes in the physicochemical properties of GelMA hydrogels, which would be valuable when attempting to interpret the complex biomaterial-tissue interactions.

## 5 Conclusion

In summary, GelMA is a versatile semi-synthetic biomaterial that exhibits detectable autofluorescence primarily at green emission wavelength ranges using 840 nm two-photon excitation. Although the photocrosslinks of GelMA are non-fluorescent, autofluorescence lifetime of the hydrogels was shortened following an increase in crosslinking levels. Moreover, through implementation of a tri-exponential decay model, it was revealed that the reduction in lifetime was due to a decrease in the presence of the long-lifetime component (A_3_). By contrast, higher pHs in GelMA hydrogels led to a decrease in mean lifetime due to greater contribution of a short lifetime component (A_1_). Importantly, our methodology was able to separately distinguish between different degrees of crosslinking and pHs without the need for exogenous sources of fluorescence. Future studies may benefit from the integration of FTIR analysis as it may provide structural insights into the collagen conformational changes that may be associated to changes in lifetime. In addition, future work will also focus on investigating the interaction between extracellular pH and GelMA lifetime as well as the role of lifetime in monitoring *in vivo* gel degradation. This may require spectral unmixing given that endogenous fluorescence emanating from cellular flavins may overlap with GelMA autofluorescence. Follow up studies may also examine the ability of FLIM to monitor the two-photon polymerization of GelMA for real-time feedback of crosslinking levels (Brigo et al., 2017; Wang et al., 2018; Atry et al., 2020; Prina et al., 2020; Sanjuan-Alberte et al., 2021; Van Der Sanden et al., 2021). The current study demonstrates that FLIM has value in non-invasively characterizing GelMA hydrogrel properties as the use of exogenous fluorescent labels and dyes for characterization of biomaterials can complicate clinical translation. Moreover, utilizing the autofluorescence of gelatin to monitor hydrogel properties through FLIM has broad applicability in regenerative medicine as it allows for visualization and quantification of tissue-material interactions without the interference of exogenous fluorophores.

## Data Availability

The raw data supporting the conclusions of this article will be made available by the authors, without undue reservation.
